# Imported Genotype 2B Rubella Virus Caused the 2012 Outbreak in Anqing City, China

**DOI:** 10.1371/journal.pone.0139173

**Published:** 2015-09-24

**Authors:** Zhen Zhu, Guixia Pan, Shujie Zhou, Jingjing Dai, Xia Chen, Jihai Tang, Shuping Chen, Yilun Zheng, Jie Song, Wenbo Xu

**Affiliations:** 1 WHO WPRO Regional Reference Measles/Rubella Laboratory and Key Laboratory of Medical Virology Ministry of Health, National Institute for Viral Disease Control and Prevention, Chinese Center for Disease Control and Prevention, Beijing, People’s Republic of China; 2 Anqing Prefecture Center for Diseases Control and Prevention, Anqing, People’s Republic of China; 3 Anhui Provincial Center for Disease Control and Prevention, Hefei, People’s Republic of China; 4 School of Medical, Anhui University of Science & Technology, Huainan, People’s Republic of China; 5 The Second Hospital of Jilin University, Changchun, People’s Republic of China; University of Hong Kong, HONG KONG

## Abstract

A rubella outbreak occurred in Anqing city of Anhui province, China, from February to July of 2012, and a total of 241 clinically diagnosed or lab-confirmed patients were reported. The highest number of rubella cases during this outbreak was recorded in teenagers between 10 and 19 years of age who had not previously received the rubella vaccine. Genotyping results indicated that the genotype 2B rubella virus (RV) was responsible for the outbreak. However, a phylogenetic analysis showed that the genotype 2B RVs isolated in Anqing City were not related to 2B RVs found in other cities of Anhui province and in other provinces of China, thus providing evidence for importation. After importation, the transmission of Anqing RVs was interrupted owing to an effective immunization campaign against rubella, suggesting the timeliness and effectiveness of contingency vaccination. Strengthening rubella surveillance, including the integration of epidemiologic information and laboratory data, is a vital strategy for rubella control and elimination. In addition, except for routine immunization, targeted supplementary immunization activities aimed at susceptible groups according to sero-epidemiological surveillance data also play a key role in stopping the continuous transmission of rubella viruses and in preventing further congenital rubella syndrome cases.

## Introduction

Rubella virus (RV) is a single-stranded, positive-sense RNA virus in the family *Togaviridae*, genus *Rubivirus* [1]. RV infections usually cause a mild, self-limiting disease. However, an infection that occurs during the first trimester of pregnancy may lead to fetal death, miscarriage, or congenital rubella syndrome (CRS) [1]. Based on the systematic RV nomenclature established by the World Health Organization (WHO), a fragment of at least 739 nt within the E1 gene is required for genotype identification and can be used to study viral transmission patterns [2]. To date, the WHO recognizes 12 RV genotypes, 1B, 1C, 1D, 1E, 1F, 1G, 1H, 1I, 1J, 2A, 2B, and 2C, and 1 provisional genotype, 1a [3]. Among them, genotypes 1E and 2B have wide geographic distributions [3, 4].

Chinese rubella virological surveillance was initiated in 1999. Over the past 14 years, a total of three wild-type RV genotypes, 1E, 1F, and 2B, have been identified in China [5–7]. The RV genotype 1F was reported in 2002; it was considered inactive and probably extinct [8]. Genotype 1E, which has been confirmed as the most frequent genotype in China, has circulated continuously since its first isolation in 2001 [7]. However, the epidemic pattern of genotype 2B in China is different from that of genotype 1E. Before 2010, the detection of genotype 2B viruses was infrequent; they were only found in 2000 and 2008 [6]. Since 2011, perhaps owing to multiple introductions, a new lineage of the 2B virus has become endemic to the mainland of China [7].

Immunization with live attenuated RV vaccine can effectively prevent infection [9]. By 2012, 132 countries introduced at least one dose of rubella-containing vaccine [10]. However, rubella remains an important pathogen and outbreaks continue to occur owing to an increased number of susceptible individuals, and rubella is listed as a category C notifiable infectious disease in China. To control rubella epidemics and prevent CRS, routine rubella vaccine was introduced into Chinese national immunization program in 2008 [7]. However, national rubella and CRS surveillance has not yet been established; thus, there is a lack of detailed information on population coverage of rubella vaccine. Here, we report a rubella outbreak caused by the RV 2B genotype in Anqing city of Anhui province, China, from February to July of 2012. The main objective of this study was to describe the epidemiological profile of this outbreak and characterize the viruses involved.

## Materials and Methods

### Ethics statement

This study did not involve human experimentation; the only human material used in this study was throat swab specimens collected from suspected rubella cases during an outbreak in Anqing city of Anhui province, China, from February to July of 2012. This study was approved by the second session of the Ethics Review Committee of the National Institute for Viral Disease Control and Prevention of the Center for Disease Control and Prevention (CDC) in China. Written informed consent for the use of clinical specimens was obtained from all patients involved in this study or their guardians.

### Specimen collection and detection

EPI (Expanded Program on Immunization) staffs from the Anqing CDC collected serum and throat swab specimens from clinically diagnosed rubella cases within 5 days after rash onset during the outbreak investigation. All samples were transported to the Anhui CDC at 4°C for laboratory diagnosis. In order to confirm the pathogens of the suspected cases, both measles and rubella IgM were detected by commercial ELISA (enzyme-linked immunosorbent assay) kits (Virion/Serion GmbH, Wurzburg, Germany).

### Virus isolation and identification

Throat swabs specimens were inoculated into Vero/Slam cells as previously described [11]. Due to few cytopathic effects in the cell cultures, the culture supernatant was harvested directly after 7 days and used to inoculate fresh cells for up to two additional passages. RNA was extracted from the cultured viruses using the QIAamp Viral RNA Extraction Mini Kit (Qiagen, Beijing, China) following the manufacturer’s instructions. One-step real-time reverse transcription-polymerase chain reaction (RT-PCR) was performed to detect viral RNA using previously described methods [12]. The amplification was carried out with an ABI 7500 Real-Time System (Life Technologies, NY, USA) and data were analyzed with 7500 software (version 2.0.1).

### Genotyping window amplification and determination

After real-time RT-PCR identification, positive samples were used to amplify a 945-nt product containing the 739-nt genotyping sequence window (nt 8,731 to 9,469) with the Qiagen One Step RT-PCR Kit according to the protocol of the US CDC [13]. After purification of the PCR product with the QIA Gel Extraction Kit (Qiagen), the amplicon sequences were determined bi-directionally using the Sanger dideoxy terminator sequencing method with a BigDye Terminator version 3.1 chemistry (Life Technologies) and the ABI PRISM 3130 DNA Sequencer (Hitachi, Japan). Sequences were edited and assembled to obtain the 739-nt fragments using Sequencher version 5.0 (Gene Codes Corporation, Ann Arbor, MI, USA). Sequence alignments and a phylogenetic analysis were performed using MEGA version 5.0.3 [14]. The accuracy of the groupings was assessed using the bootstrap method with 1,000 replicates. The nucleotide sequences of 27 RV strains isolated in this outbreak and 21 RV strains from eight other cities in Anhui province during 2012–2014 are available in the GenBank database under accession numbers KJ684004, KJ684044, and KT160437-KT160482. An additional 14 sequences of RV genotype 2B from 11 other provinces in the mainland of China and 50 sequences of genotype 2B from 14 other countries/regions outside of China were retrieved from the GenBank database (S1 Table) for further phylogenetic analysis to understand the origin and transmission of RV genotype 2B, which was responsible for this outbreak.

## Results

### Epidemiological profile of the 2012 outbreak

**Fig 1:**
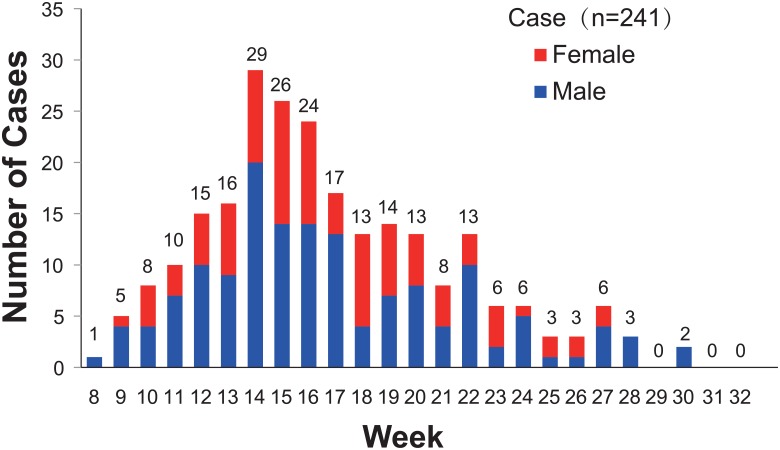
Reported rubella cases by week of onset in Anqing city of Anhui province in 2012.

**Fig 2:**
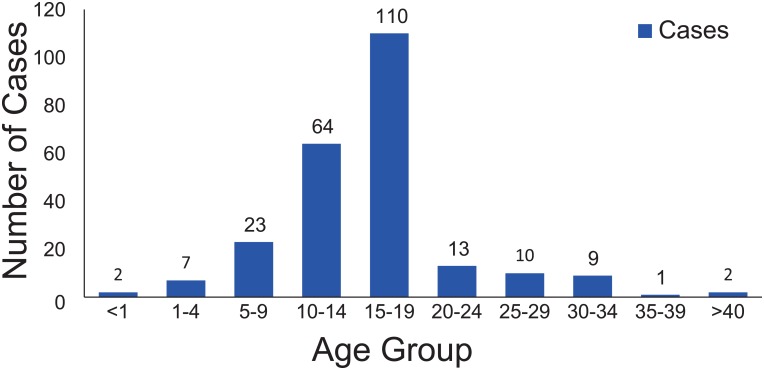
Number of reported rubella cases by age group.

**Fig 3:**
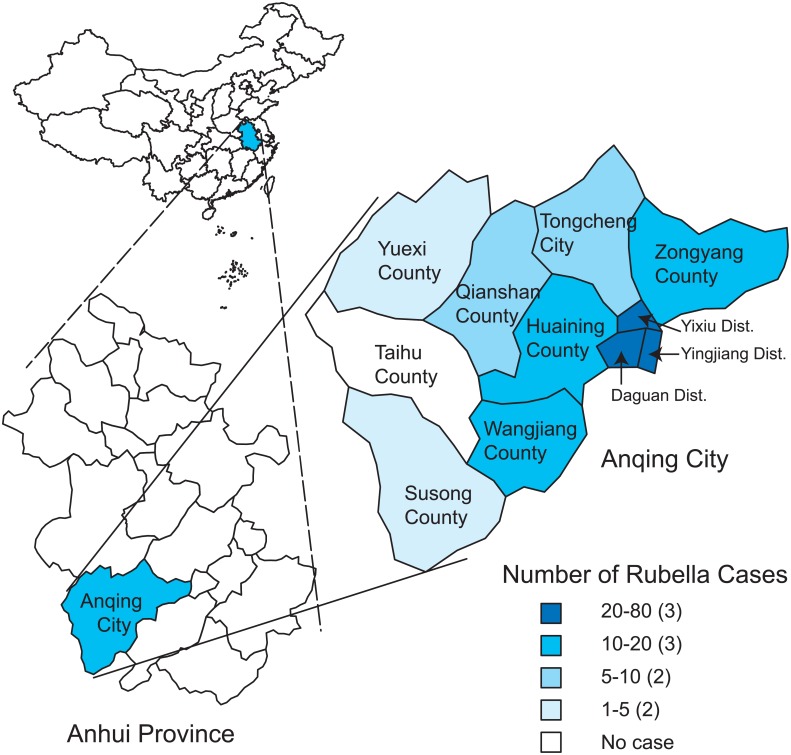
The geographic distribution of rubella cases in Anqing City, Anhui province, China.

On February 25, 2012, a junior high school student from a foreign language school located in the Yixiu district of Anqing city in Anhui province, China, experienced fever and rash and sought treatment in a health center near the school. Rubella was confirmed based on rubella-specific IgM detection, but the detailed epidemiological information regarding this case is unknown. Subsequently, the number of rubella cases in Anqing gradually increased, and the incidence of relevant cases peaked from the 14th to the 16th week in 2012. By July 26, 2012, a total of 241 clinically diagnosed or lab-confirmed patients were reported to the National Notifiable Disease Reporting System (NNDRS, http://10.249.1.170/) (Fig 1) of the China CDC, and more than 80% of these were lab-confirmed cases. This rubella outbreak mainly affected students aged 10–19 years (72.2%) who had not received a rubella vaccine (Fig 2). The overall male to female ratio was 1.6:1. Anqing, located in southwest Anhui province, is composed of 3 districts, 7 counties, and 1 county-level city. Geographically, the rubella cases were distributed across all administrative areas of Anqing, except for Taihu County. The majority of cases (77.6%) were reported from Daguan (73 cases), Yingjiang (62 cases), and Yixiu Districts (52 cases), which are located in the eastern part of the city, and the epidemic spread westward. Susong (1 case) and Yuexi counties (2 cases), located in the western part of the city, had the fewest cases (Fig 3). To interrupt viral transmission, an immunization campaign against rubella was carried out among the citywide school students at the beginning of April. Subsequently, the number of rubella cases dramatically decreased to zero, suggesting the timeliness and effectiveness of contingency vaccination.

### Genotype 2B RVs responsible for the outbreak

A total of 27 RVs were isolated from throat swabs during the outbreak. These viruses were distributed across all 3 districts [Yixiu (10), Yinjiang (5), and Daguan (5)], 6 counties [Zongyang (1), Huaining (1), Wangjiang (1), Qianshan (1), Susong (1), and Yuexi (1)] and 1 county-level city [Tongcheng (1)]. A sequence analysis of the 739-nt region within the E1 gene revealed almost identical sequences (<0.41% nucleotide difference), indicating a single chain of transmission, and all the patients were infected by the same virus. All RVs were named based on WHO guidelines [3].

**Fig 4:**
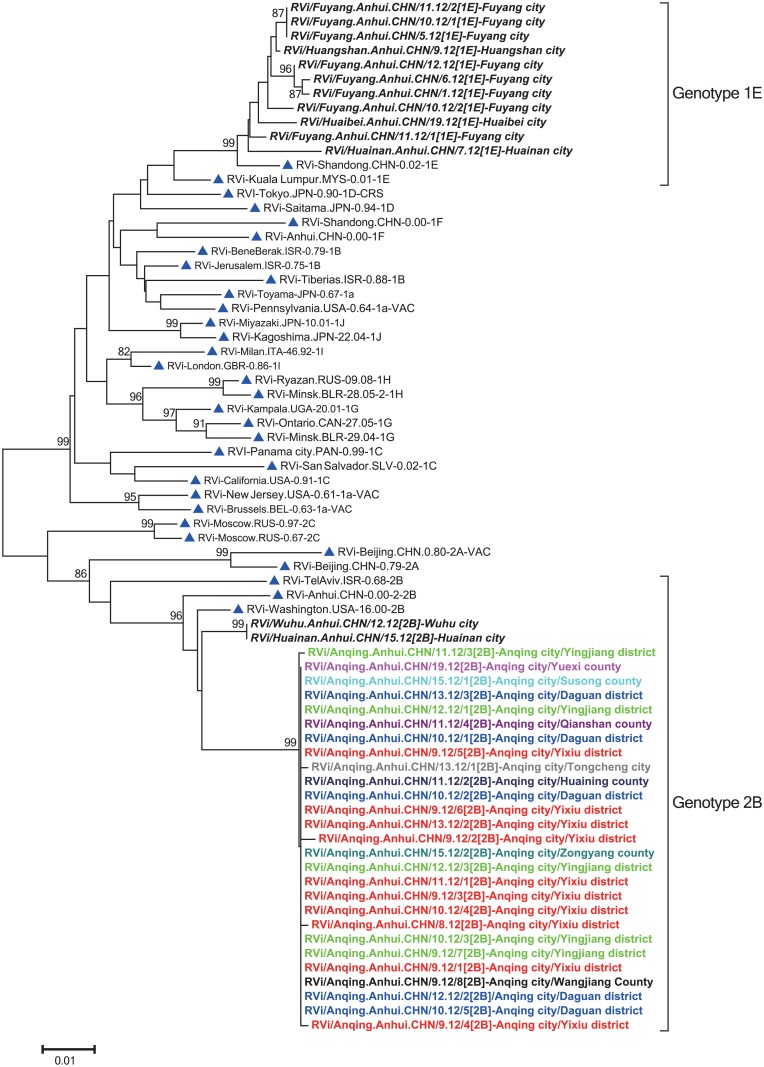
Phylogenetic analysis of 27 rubella virus (RV) sequences from the outbreak in Anqing city and 13 RVs from other cities in Anhui province in 2012, as well as the World Health Organization (WHO) reference sequences based on the WHO standard 739-nt window. The 32 WHO reference strains are indicated by blue triangles. The viruses from other cities are indicated by bold italic font. The viruses from 6 counties, 3 districts, and 1 county-level city of Anqing outbreak are indicated by different colors; and the first case of the outbreak is indicated by a red circle.

A phylogenetic analysis of the 739-nt sequences of 27 RVs from the outbreak in Anqing, 13 RVs from another 5 cities in Anhui province collected in the same year [Fuyang (8), Huaibei (1), Huainan (2), Wuhu (1), and Huangshan (1)], and WHO reference viruses showed that these 40 Anhui RV sequences could be divided into 2 genotypes, 1E and 2B. The 27 Anqing RVs, which differed from the RVs of genotype 2B collected in other cities (Wuhu and Huainan city), formed an independent lineage within genotype 2B with high bootstrap support. Thus, the results suggested that genotype 2B RVs responsible for the outbreak and Anqing RVs were different from RVs circulating in other regions of Anhui province in 2012 (Fig 4).

### Phylogenetic analysis indicated that genotype 2B RV isolated in Anqing was imported

**Fig 5:**
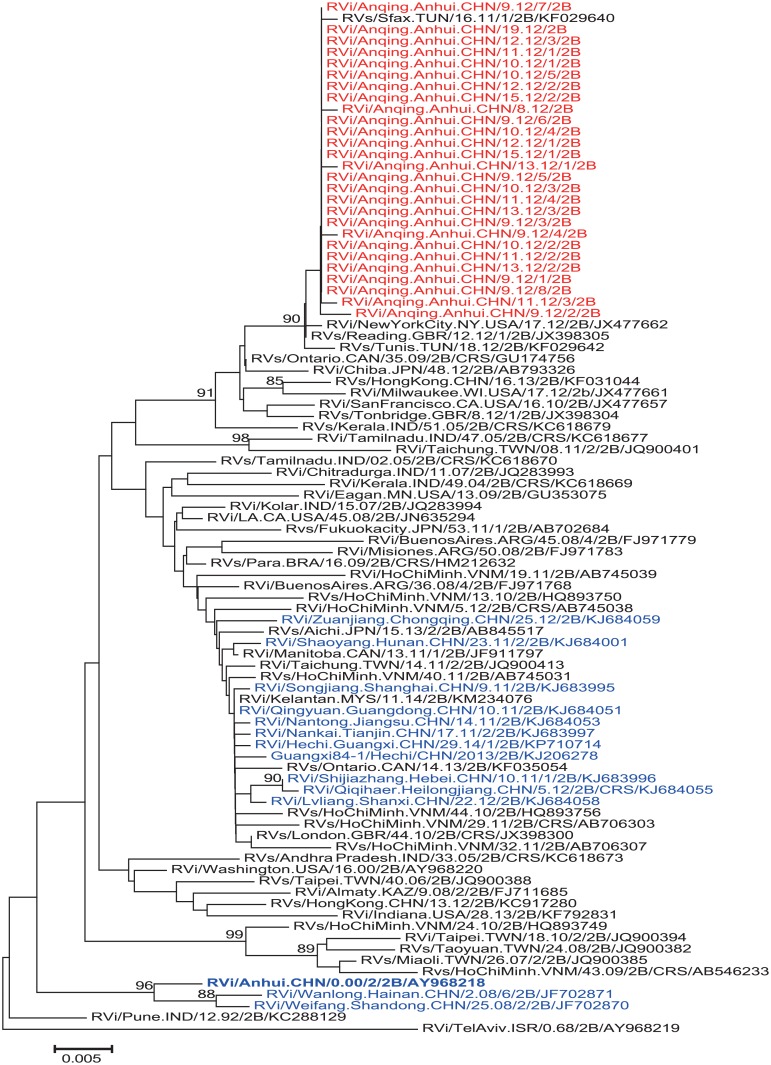
Phylogenetic analysis of the sequences of 27 RVs from the outbreak in Anqing city (shown in red), 14 domestic genotype 2B RVs (2000–2014) (shown in blue), and 50 international genotype 2B RVs (1968–2014) downloaded from the GenBank database based on the WHO standard 739-nt window.

To investigate the source of the Anqing RVs, a phylogenetic analysis was conducted on the basis of the 739-nt region of 14 domestic genotype 2B RVs (2000–2014) downloaded from the GenBank database and 50 international genotype 2B RVs (1968–2014) including those from Vietnam (9), India (8), United States of America (7), Taiwan (6), United Kingdom (3), Canada (3), Japan (3), Argentina (3), Hong Kong (2), Tunisia (2), Malaysia (1), Brazil (1), Kazakhstan (1), and Israel (1) (Fig 5). The results indicated that Anqing RVs were closely related to an RV from Tunisia in 2011 (strain RVs/Sfax.TUN/16.11/1/2B, GenBank accession no. KF029640), exhibiting the highest nucleotide sequence identity (99.5%–99.8%). In addition, Anqing RVs also clustered with RVs from the United Kingdom isolated in 2012 (strain RVs/Reading.GBR/12.12/1; identity: 99.5%–99.8%; GenBank accession number JX398305) and the United States of America isolated in 2012 (Strain RVi/NewYorkCity.NY.USA/17.12; identity: 99.4%–99.7%; GenBank accession number JX477662; imported from Italy based on GenBank information), with high bootstrap support (90%). These results revealed that the Anqing genotype 2B RVs probably originated from North African or European countries.

**Fig 6:**
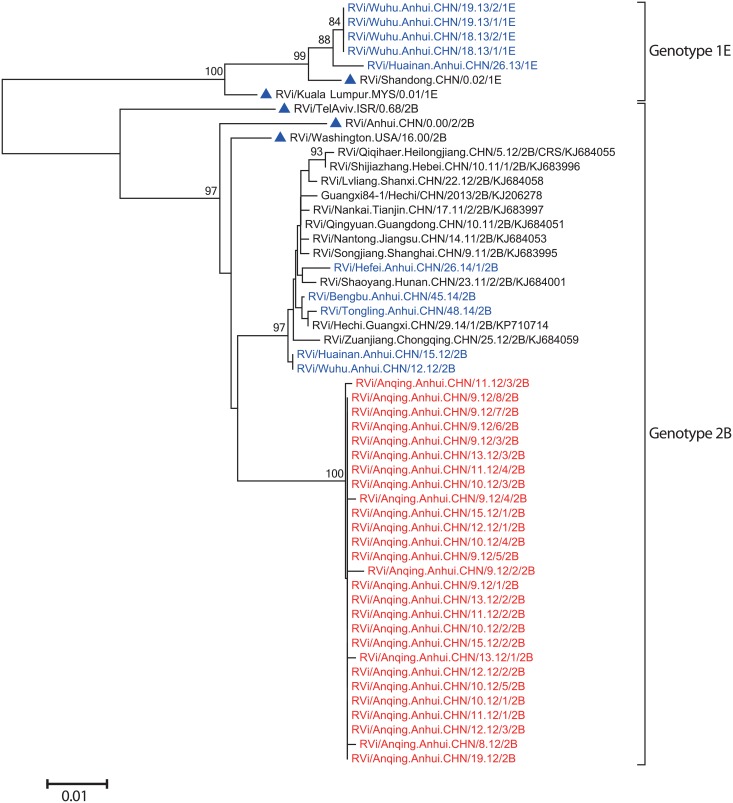
Phylogenetic analysis of the sequences of 27 Anqing RVs (shown in red), 10 RVs collected in 5 cities of Anhui province between 2012 and 2014 (shown in blue), and 11 2B RVs from other provinces of China during 2011–2014 based on the WHO standard 739-nt window. Genotype 1E and 2B WHO reference strains are indicated by blue triangles.

To further understand the transmission of Anqing RVs in Anhui province since 2012, a phylogenetic analysis of Anqing RV sequences, 10 RVs collected in 5 cities of Anhui province between 2012 and 2014 [Wuhu (2012:1; 2013:4), Huainan (2012:1; 2013:1), Bengbu (2014:1), Tongling (2014:1), and Hefei (2014:1)], and 11 2B RVs from other provinces of China collected during 2011–2014 (Fig 6) showed that RVs in Anhui province in 2013 and 2014 could be grouped into genotype 1E (5 RVs from 2 cities in 2013) and 2B (3 RVs from 3 cities in 2014), respectively. Genotype 2B RVs in 2014 clustered separately from all Anqing RVs, and were closely related to 2B RVs from Wuhu and Huainan city in 2012 and from provinces of China other than Anhui, the currently predominant virus circulating in China (unpublished data). These data indicated that after importation, the transmission of Anqing RVs was likely interrupted owing to effective rubella vaccination.

## Discussion

The important genetic baseline spanning 14 years in mainland China has been established, and co-circulation of genotypes 1E and 2B has been found in recent years [7]. The results of the present study indicated that genotype 2B RV was responsible for the 2012 outbreak in Anqing city in Anhui province of China. However, based on the phylogenetic tree, the genotype 2B RVs isolated in Anqing City located in different lineages of 2B RVs that were found in other cities of Anhui province and in other provinces of China, which should be considered potential sources of importation.

Due to the limited epidemiological data, the source of the virus was difficult to identify. Furthermore, due to the lack of comprehensive rubella surveillance data in many regions worldwide, sufficient geographic diversity representing the global genotype distribution was unavailable, making it impossible to trace the RV transmission pattern. WHO data showed that few countries with rubella cases reported genotype information (www.who.int); therefore, in order to support rubella control and elimination programs, sustained efforts to strengthen rubella virological surveillance worldwide should be improved, especially in those countries where rubella virological surveillance is not well established.

RV is a candidate for worldwide eradication [10], but numerous RV outbreaks have recently been reported in many countries [15–19]. This study emphasized that following the confirmation of the etiology of an outbreak, measures such as emergency catch-up campaigns are very useful and important to protect susceptible individuals and to stop further viral transmission. These measures have also been effective for preventing further spread of outbreaks in other countries [15]. In addition, since the laboratory plays a crucial role in providing timely and accurate confirmation of suspected rubella cases, distinguishing between imported and endemic rubella cases, and tracing RV transmission patterns through sequence analyses [20], strengthening rubella surveillance via the integration of epidemiologic information and laboratory data is a vital strategy to accelerate the control and elimination of rubella.

Rubella vaccine was introduced into the national immunization program in China in 2008 [7]. According to the national immunization strategy, a two-dose vaccine schedule is administrated to infants at 8 months of age (Measles-Rubella vaccine, MR) and at 18–24 months of age (Measles-Mumps-Rubella vaccine, MMR) [7]. However, the highest number of rubella cases during this outbreak was recorded in teenagers between 10 and 19 years old, reflecting the incompleteness of the current vaccine immunization strategy to provide rubella-containing vaccine to infants in China. Moreover, continuous epidemiological surveillance data from Beijing City and Shandong Province of China indicates that the disease burden has shifted to an older age group (15- to 39-year-old individuals) [13, 21]. Another lesson came from the rubella epidemic between 2012–2013 in Japan, the reemergence of rubella mainly affected adult men aged 35–51 years, who had not received routine rubella vaccine during their childhood when only school girls were vaccinated [22]. Hence, in addition to the routine rubella vaccine immunization program, sero-epidemiological surveillance is crucial to determine the prevalence of rubella antibodies in different populations and geographical regions, and targeted supplementary immunization activities aimed at susceptible groups can be implemented to stop the continuous transmission of the virus and prevent further CRS cases; otherwise, rubella control in China may be impeded.

## Supporting Information

S1 Table List of rubella virus sequences used for the analysis. (DOCX)
