# Supplementary material for: Imported Genotype 2B Rubella Virus Caused the 2012 Outbreak in Anqing City, China
Source: PLoS One. 2015 Sep 24;10(9):e0139173. doi: 10.1371/journal.pone.0139173 (PMC4581689; doi:10.1371/journal.pone.0139173)
Supplement: S1 Table — (DOCX) [file pone.0139173.s001.docx]

**S1 Table. List of rubella virus sequences used for the analysis.**

| **Strain name** | **Country/Region** | **Year of isolation** | **GenBank accession number** |
| --- | --- | --- | --- |
| RVi/TelAviv.ISR/0.68/2B | Israel | 1968 | AY968219 |
| RVi/Pune.IND/12.92/2B | India | 1992 | KC288129 |
| RVi/Washington.USA/16.00/2B | United States of America | 2000 | AY968220 |
| RVi/Anhui.CHN/0.00/2/2B | China | 2000 | AY968218 |
| RVi/Kerala.IND/49.04/2B/CRS | India | 2004 | KC618669 |
| RVs/Kerala.IND/51.05/2B/CRS | India | 2005 | KC618679 |
| RVi/Tamilnadu.IND/47.05/2B/CRS | India | 2005 | KC618677 |
| RVs/Tamilnadu.IND/02.05/2B/CRS | India | 2005 | KC618670 |
| RVs/Andhra Pradesh.IND/33.05/2B/CRS | India | 2005 | KC618673 |
| RVs/Taipei.TWN/40.06/2B | Taiwan | 2006 | JQ900388 |
| RVi/Chitradurga.IND/11.07/2B | India | 2007 | JQ283993 |
| RVi/Kolar.IND/15.07/2B | India | 2007 | JQ283994 |
| RVs/Miaoli.TWN/26.07/2/2B | Taiwan | 2007 | JQ900385 |
| RVi/LA.CA.USA/45.08/2B | United States of America | 2008 | JN635294 |
| RVi/BuenosAires.ARG/45.08/4/2B | Argentina | 2008 | FJ971779 |
| RVi/Misiones.ARG/50.08/2B | Argentina | 2008 | FJ971783 |
| RVi/BuenosAires.ARG/36.08/4/2B | Argentina | 2008 | FJ971768 |
| RVi/Almaty.KAZ/9.08/2/2B | Kazakhstan | 2008 | FJ711685 |
| RVi/Wanlong.Hainan.CHN/2.08/6/2B | China | 2008 | JF702871 |
| RVi/Weifang.Shandong.CHN/25.08/2/2B | China | 2008 | JF702870 |
| RVs/Taoyuan.TWN/24.08/2B | Taiwan | 2008 | JQ900382 |
| Rvs/HoChiMinh.VNM/43.09/2B/CRS | Vietnam | 2009 | AB546233 |
| RVs/Ontario.CAN/35.09/2B/CRS | Canada | 2009 | GU174756 |
| RVi/Eagan.MN.USA/13.09/2B | United States of America | 2009 | GU353075 |
| RVs/Para.BRA/16.09/2B/CRS | Brazil | 2009 | HM212632 |
| RVi/SanFrancisco.CA.USA/16.10/2B | United States of America | 2010 | JX477657 |
| RVs/HoChiMinh.VNM/13.10/2B | Vietnam | 2010 | HQ893750 |
| RVs/HoChiMinh.VNM/44.10/2B | Vietnam | 2010 | HQ893756 |
| RVs/London.GBR/44.10/2B/CRS | United Kingdom | 2010 | JX398300 |
| RVs/HoChiMinh.VNM/24.10/2B | Vietnam | 2010 | HQ893749 |
| RVi/Taipei.TWN/18.10/2/2B | Taiwan | 2010 | JQ900394 |
| RVs/Sfax.TUN/16.11/1/2B | Tunisia | 2011 | KF029640 |
| RVi/Taichung.TWN/08.11/2/2B | Taiwan | 2011 | JQ900401 |
| Rvs/Fukuokacity.JPN/53.11/1/2B | Japan | 2011 | AB702684 |
| RVi/HoChiMinh.VNM/19.11/2B | Vietnam | 2011 | AB745039 |
| RVi/Manitoba.CAN/13.11/1/2B | Canada | 2011 | JF911797 |
| RVi/Taichung.TWN/14.11/2/2B | Taiwan | 2011 | JQ900413 |
| RVs/HoChiMinh.VNM/40.11/2B | Vietnam | 2011 | AB745031 |
| RVi/Shaoyang.Hunan.CHN/23.11/2/2B | China | 2011 | KJ684001 |
| RVi/Songjiang.Shanghai.CHN/9.11/2B | China | 2011 | KJ683995 |
| RVi/Qingyuan.Guangdong.CHN/10.11/2B | China | 2011 | KJ684051 |
| RVi/Nantong.Jiangsu.CHN/14.11/2B | China | 2011 | KJ684053 |
| RVi/Nankai.Tianjin.CHN/17.11/2/2B | China | 2011 | KJ683997 |
| RVi/Hechi.Guangxi.CHN/29.14/1/2B | China | 2011 | KP710714 |
| RVi/Shijiazhang.Hebei.CHN/10.11/1/2B | China | 2011 | KJ683996 |
| RVs/HoChiMinh.VNM/29.11/2B/CRS | Vietnam | 2011 | AB706303 |
| RVs/HoChiMinh.VNM/32.11/2B | Vietnam | 2011 | AB706307 |
| RVs/Reading.GBR/12.12/1/2B | United Kingdom | 2012 | JX398305 |
| RVi/NewYorkCity.NY.USA/17.12/2B | United States of America | 2012 | JX477662 |
| RVs/Tunis.TUN/18.12/2B | Tunisia | 2012 | KF029642 |
| RVi/Chiba.JPN/48.12/2B | Japan | 2012 | AB793326 |
| RVi/Milwaukee.WI.USA/17.12/2B | United States of America | 2012 | JX477661 |
| RVs/Tonbridge.GBR/8.12/1/2B | United Kingdom | 2012 | JX398304 |
| RVi/HoChiMinh.VNM/5.12/2B/CRS | Vietnam | 2012 | AB745038 |
| RVi/Zuanjiang.Chongqing.CHN/25.12/2B | China | 2012 | KJ684059 |
| RVi/Qiqihaer.Heilongjiang.CHN/5.12/2B/CRS | China | 2012 | KJ684055 |
| RVi/Lvliang.Shanxi.CHN/22.12/2B | China | 2012 | KJ684058 |
| Guangxi84-1/Hechi/CHN/2013/2B | China | 2013 | KJ206278 |
| RVs/HongKong.CHN/13.12/2B | Hongkong, China | 2012 | KC917280 |
| RVs/Aichi.JPN/15.13/2/2B | Japan | 2013 | AB845517 |
| RVs/Ontario.CAN/14.13/2B | Canada | 2013 | KF035054 |
| RVs/HongKong.CHN/16.13/2B | Hongkong, China | 2013 | KF031044 |
| RVi/Indiana.USA/28.13/2B | United States of America | 2013 | KF792831 |
| RVi/Kelantan.MYS/11.14/2B | Malaysia | 2014 | KM234076 |
